# Design of a Wireless Sensor System with the Algorithms of Heart Rate and Agility Index for Athlete Evaluation

**DOI:** 10.3390/s17102373

**Published:** 2017-10-17

**Authors:** Meina Li, Youn Tae Kim

**Affiliations:** 1 College of Instrumentation and Electrical Engineering, Jilin University, Changchun 130061, China; 2 Department of IT Fusion Technology, Graduate School, Chosun University, Gwangju 61452, Korea

**Keywords:** heart rate detection, agility index, athlete evaluation

## Abstract

Athlete evaluation systems can effectively monitor daily training and boost performance to reduce injuries. Conventional heart-rate measurement systems can be easily affected by artifact movement, especially in the case of athletes. Significant noise can be generated owing to high-intensity activities. To improve the comfort for athletes and the accuracy of monitoring, we have proposed to combine robust heart rate and agility index monitoring algorithms into a small, light, and single node. A band-pass-filter-based R-wave detection algorithm was developed. The agility index was calculated by preprocessing with band-pass filtering and employing the zero-crossing detection method. The evaluation was conducted under both laboratory and field environments to verify the accuracy and reliability of the algorithm. The heart rate and agility index measurements can be wirelessly transmitted to a personal computer in real time by the ZigBee telecommunication system. The results show that the error rate of measurement of the heart rate is within 2%, which is comparable with that of the traditional wired measurement method. The sensitivity of the agility index, which could be distinguished as the activity speed, changed slightly. Thus, we confirmed that the developed algorithm could be used in an effective and safe exercise-evaluation system for athletes.

## 1. Introduction

The heartbeat is a vital physical parameter for the evaluation of health conditions, as it provides the vital signs of heart functions. The morphologies and inter-beat intervals of electrocardiogram (ECG) waveform can reveal the condition of the heart contractions. The ECG signal can be used to detect the heart rate accurately [1,2,3,4]. The QRS complex is the most prominent waveform in the ECG signal that can reveal the electrical activities during ventricular contractions. The current state of the heart can be evaluated by the shape of the QRS complex as well as by the time when it occurs. High-accuracy QRS detection is difficult because various types of noises occur in the ECG signal. Muscle action, electrode motion, and baseline instability can be the sources of noise [5]. A typical example is the loss of contact between the electrode and skin, which causes a transient interference in the baseline; this is called the electrode-contact noise [6]. There are some early studies for estimating heart rate such as by using acoustic [7] and Photoplethysmography (PPG) sensors [8,9,10]. The technology of the PPG signals relies on data from irradiating a light-emitting diode (LED) towards the capillary vessels in the skin and receiving the reflected light by a photo detector. These measures sense the rate of blood flow as controlled by the heart pumping action. Fukushima et al. [11] suggested a spectrum subtraction method to reject the spectrum of acceleration data from PPG signals. This method was proposed for the scenarios when small movements were incurred. For athletes, vigorous movements and vibrations are significant sources of noise. The motion artifacts are the transient baseline caused by changes in the electrode-skin impedance [12,13,14]. This is an especially relevant issue for athletes engaged in high-intensity activities. Unexpected noise with random amplitude and frequency can easily cause false detection. In addition, the physiological variability of the QRS complex makes heart rate detection more difficult. The occurrence of T waves with high-frequency characteristics, which are similar to those of the QRS complex, can hinder QRS detection. The accuracy of heart rate monitoring is not high during intensive physical activity, which means that it is difficult to determine the heart rate accurately during an emergency by just analyzing heart stress. The other novel TROIKA framework was proposed by Zhange et al. [9]. This method was based on signal decomposition, sparse signal reconstruction, and spectral peak tracking for versification. However, this approach is computationally demanding and may not work on real-time fitness devices. Due to the limitations stated above, these proposed methods might not be effective when subjects perform intensive exercise.

In sports, agility can indicate the potential of an athlete. To improve agility, many training exercises have been proposed [15,16,17]. The measurement of agility is based on the time that an athlete needs to complete the prescribed exercise or how many steps they can complete in a given period. It is well known that the intensity of body movement has a close relationship with the physical demand of the human circulatory system. The agility index is manually calculated by a supervisor. The manual measurement, calculation, and analysis of the agility index are inconvenient and inaccurate. Human motion analysis has been primarily dominated by the video camera [18]. L. Sigal et al. digitized the captured video of motion states, and the movements can be enhanced for further analysis by motion analysis software. B. Dijkstra et al. [19] monitored physical activities with accelerometers and compared them with video observations. T. Ryan Burchfield et al. [20] developed a wireless sensor application that detects abnormal human movements, such as seizures. In another study, a kinematic sensor [21] composed of a multi-axial accelerometer and gyroscope was used to monitor the daily physical activities of elderly people. J. Ng et al. [22] developed a device that could record information on body positions along with the ECG signal to aid clinical diagnosis. However, motion detection with multiple cameras requires a complicated setup in a limited space, and therefore, it is unsuitable for monitoring people in outdoor or mobile environments. The aforementioned studies, which were related to body posture, were limited by the accuracy of the system. For a more accurate depiction of body posture and position, it would be necessary to mount more sensors at various locations on the body.

There are commercially available products in the market. Usually, they are watch-type and belt-type that strap the sensor on the chest. The other commercial product is the pedometer that uses an accelerometer. These devices usually have a single function, such as heart rate or movement monitoring. The watch-type sensor has high accuracy in monitoring walking and running, but it does not have a high correlation with heart rate. The watch-type sensor may not tightly attach to the skin during intensive exercise. Further, the belt-type also needs to be worn tightly on the chest, which is not comfortable during running. It may easily drop during running due to profuse sweating. Other research has been done on smart garments such as the ATHOS suit, Polo Tech, and Medical smart garments. These wearable devices provide healthcare solutions. However, since the shape of each user's chest is different and the electrodes are stitched on the chest area, they cannot be accurately attached. If there is a disturbance due to violent motion, noise is generated and the accuracy is greatly reduced. Hence, the garments would need to be specially designed to be accurate for individuals.

Therefore, we have designed our small patch-type sensor for athletes. It can monitor both heart rate and movement simultaneously, and it is much more comfortable for the athlete. It is also unobtrusive. The data can be recorded and downloaded for further analysis. Our study aimed to provide an appropriate and highly accurate method for heart rate monitoring under harsh conditions, especially those that athletes encounter. Further, we aimed to develop a new indicator that correlates well with the conventional agility index, and focus on the development of a small, lightweight sensor module to collect vital information, because the patch-type sensor, with its considerable weight, can enhance the effect of electrode-contact noise. The agility index can effectively respond to the changing body position, which gives information on speed and strength. The patch-type sensor averages the triangular areas of accelerations per second as a new indicator that denotes agility and can be used to evaluate exercise performance as well as athletes’ potential. The added knowledge of subjects’ activity intensity provides valuable information for monitoring, diagnostics, or health alerts. The information, which is provided by the combination of simultaneous monitoring of the heart rate and body movement intensity, comprises a valuable set of physiological and behavioral data related to physical activities and cardiac activities. Based on this information, we can observe the physical demand of the human circulatory system and the exercise performance; we can also evaluate the ability of athletes.

The rest of the paper is organized as follows. Section 2 describes in detail the algorithm for heart rate and agility index detection. Section 3 presents the overall system architecture, including the sensor board and signal processing. Section 4 evaluates the system, including the experimental results. Section 5 discusses the application to and benefits for athletic training and selection. Conclusions are presented in Section 6.

## 2. Algorithm Design

The heart rate and agility index are the two major monitoring parameters. The algorithms presented can record these in daily life as well as during high-intensity sports training. The physiological heart rate waveform is analyzed and the design of monitoring is featured by the ECG lead. The agility index is derived from three output signals of a tri-axial accelerometer. The details are provided in the following sections.

### 2.1. Algorithm for Heart Rate Monitoring

**Figure 1:**
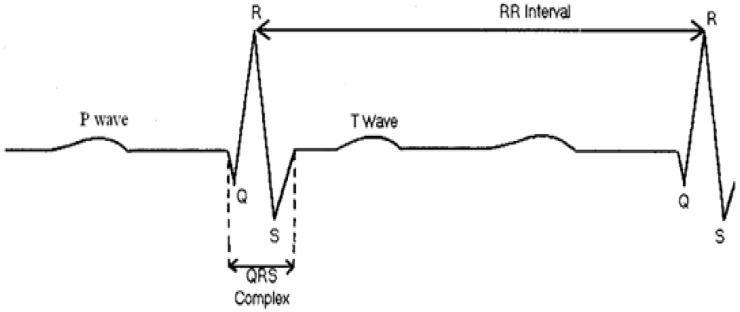
The components of an electrocardiogram (ECG) signal.

Normally, an ECG signal contains several types of waves with different periods, as shown in Figure 1. The P wave represents the initiation of the heartbeat in the upper chambers of the heart (atria), and its duration is usually less than 0.12 s [23]. The QRS complex corresponds to the lower chambers’ (ventricular) depolarization. Its duration is normally 0.04 to 0.12 s [24]. The T wave represents the recovery phase. To calculate the heart rate, the position of the QRS complex and the RR interval (the RR interval is the time that elapses between two consecutive R waves) should be precisely specified.

**Figure 2:**
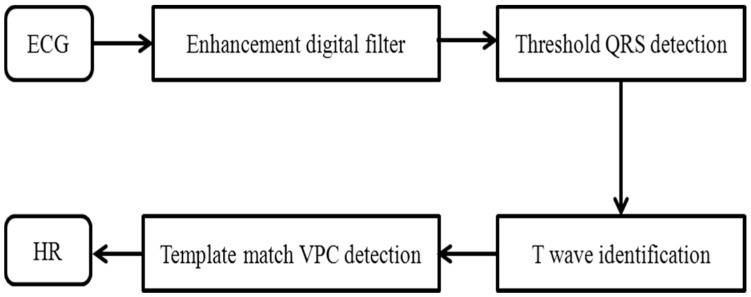
Flow chart for the heart rate detection algorithm. VPC = ventricular premature contraction; HR = heart rate.

In this study, we aimed to find an effective method that is suitable for the low processing capacity of a patch-type sensor. The algorithm for detecting the heart rate is focused on analyzing the athlete’s heart rhythm during exercise. A band-pass-filter-based R-wave detection algorithm was designed to make it more robust during motion. This algorithm acquired the Lead II signal according to the flowchart shown in Figure 2. The detection is based on the combination of digital filtering to reduce the effect of noise, threshold QRS detection, a T-wave discriminating technique to distinguish the QRS complex from the T wave, and the template-matching method to distinguish the QRS complex from the ventricular premature contraction (VPC), because QRS and VPC waveforms are similar to those of a normal contraction.

**Figure 3:**
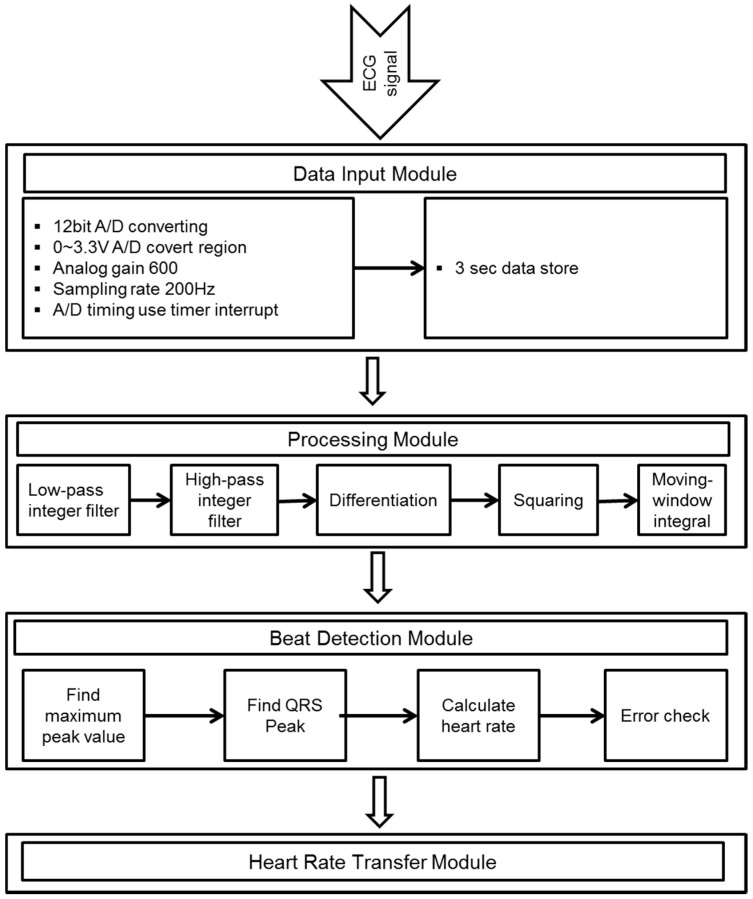
Algorithm for heart rate calculation. A/D = analog-to-digital.

Figure 3 shows the algorithm for heart rate calculation. The ECG signal is converted into a digital signal by an analog-to-digital converter (ADC) with a sampling rate of 200 Hz. The 12-bit ADC stores data for a period of 3 s to find the maximum peak value. The digitalized signal is passed through the enhancement digital filter stage to reduce the effect of noise. The filter stage includes the following processing steps. The combination of low and high-pass filters creates an effective band-pass filter. The pass band extends up to the maximum energy of the QRS complex, which is approximately 5 to 15 Hz. Use of the integer coefficient, which reduces the operation of the microcontroller by a considerable amount, real-time filtering, and other calculations to identify the QRS can be implemented in a microcontroller (MSP430F149). The differentiation step provides the QRS complex slope information. After differentiation, the signal is passed through a squaring process to enhance the slope of the frequency-response curve as well as to reduce false positives caused by the T wave with highly abnormal energy. Finally, the moving-window integration process is used to obtain slope information and the width of the QRS complex. The width (number of samples) of the moving window is very important. It should be the same as the widest QRS complex in the ECG signal. If the window is too narrow, the integrated waveform can produce several peaks. On the other hand, if the window is too wide, the QRS and the T wave can be merged to form the integrated signal. These problems can make it difficult to detect the QRS complex in the next step.

The filtered signal is passed through an adaptive-threshold QRS detection procedure to detect the QRS candidate (QRS*). Through the QRS* detection stage, thresholds are chosen and adjusted to float over the noise. Taking advantage of the high signal-to-noise ratio provided by the digital filter in the previous stage, both low and high thresholds are used. First, the higher threshold is used to detect QRS*, and if no signal is found within the predefined period, the back-searching process is conducted using the lower threshold. The details of this process are as follows:

*Finding the maximum peak value*: The input data are stored for 3 s, and then the maximum value is selected by measuring the peak-to-peak value. The high threshold is half the maximum peak value.

*Finding the QRS candidate*: The maximum value is searched in the 240-ms interval region from the first point over the threshold value.

*Comparing peak values*: A comparison with the other peak values are conducted and the QRS candidate can be considered as a QRS complex if it is the only maximum value in the interval region of 240 ms before and after it. If there is a peak value greater than the QRS candidate, the 240-ms interval region is skipped, and the candidate is removed.

*Comparing QRS intervals*: The RR interval between the determined QRS candidate and the previous QRS can be compared with the previous RR interval. If the current RR interval is too short, it indicates that the T wave or another abnormal signal has been detected. If the current RR interval is longer than 1.3 times the previous RR interval, there is a possibility of missing the QRS complex. Therefore, the back-searching process is performed.

*Performing a back-searching process*: As the back-searching process is conducted, the low threshold is fixed as 0.2 times the maximum peak value. The decision of the QRS complex is then performed as the foregoing process. After going through the earlier steps, the QRS* is decided and can be inspected again before being identified as a QRS complex by the template matching stage. The template is derived from the previously detected QRS complex. The morphologies and inter-beat intervals of the ECG signal are in the normal standard phase for most people. Different persons have different ECG morphologies, and the heartbeat too has different frequencies for different activities. Therefore, a fixed template is not used. At the beginning, the normal standard QRS complex is used as the template through the initial learning process to generate the features of the QRS complex, such as thresholds and peak value. Then, the measured signal is compared with the first QRS template. Subsequently, the QRS template is continuously updated based on the cross-correlation coefficients until the analysis of all signals is completed=. The cross-correlation coefficients calculated between the QRS* and QRS templates can be defined as

(1) $$Coe=\frac{\sum_{i=1}^{N}\left(x_{i}-X\right)\left(y_{i}-Y\right)}{\sqrt{\sum_{i=1}^{N}{\left(x_{i}-X\right)}^{2}}\sqrt{\sum_{i=1}^{N}{\left(y_{i}-Y\right)}^{2}}}$$

where *x_i_* and *y_i_* are the QRS* and the QRS templates, respectively. The coefficient has values between −1 and +1. The value that is approximately equal to 1 shows a high similarity to the QRS template. On the other hand, the lower numbers refer to VPC, which has a different waveform. To minimize the calculation, the window size N of the QRS template only spans the duration of the detected QRS*, and it is updated after each QRS is determined. In the foregoing steps, the QRS peaks are dropped if they are lower than 50% of the previous value.

### 2.2. Algorithm for Agility Index Detection

A novel agility index was developed as a measure of the sensitivity of the activity response and the amount of training for athletes. This new indicator has a high correlation coefficient with the conventional agility index. In this study, the change in acceleration during exercise is detected by using a 3-axis accelerometer (MMA7260Q) [25,26]. The sensitivity range can be adjusted at low gravity levels, or can enable more coarse-grained data acquisition at high gravity levels.

First, the acceleration signal is digitalized by using a 12-bit ADC with a 100 Hz sampling rate. The signal is not noise free; a preprocessing stage is required to reduce the effect of noise. For digital filtering, a band-pass filter and the moving average are used. For each axis, the accelerometer generates an analog voltage that corresponds to the accelerometer force parallel to that axis. The g-value of each axis is computed independently and then combined into a single value that requires less microcontroller memory and computational resources. The root mean squared formula is as follows:

(2) $$g=\sqrt[{2}]{{g_{x}}^{2}+{g_{y}}^{2}+{g_{z}}^{2}}$$

**Figure 4:**
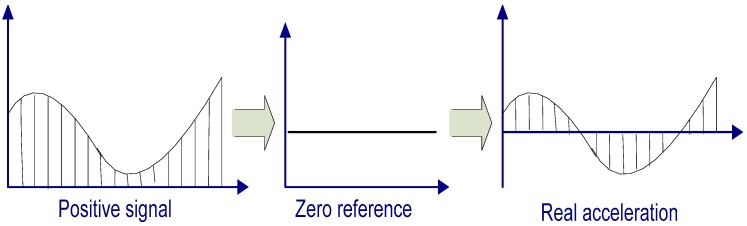
Normalization stage.

From the output characteristics of the MMA7260Q sensor, it is evident that the detected signal is always positive. However, the measured acceleration can be positive or negative, depending on the velocity change. Therefore, the filtered signal is passed through a normalization stage, which is shown in Figure 4. The maximum and minimum peak values can be determined as feature values from the accelerometer output. Finally, the area between the feature points can be calculated as the agility index.

## 3. System Architecture

The challenge for the system is to integrate all electronic chips into one sensor node with small size, light weight, low power consumption, and low cost. Most commercial sensors have a unique function and lack analysis software or data storage. Our system enables signal acquisition from multiple sensors, wireless monitoring, and display. The design of our system includes a sensor module with two proposed algorithms implemented and a portable computer with analysis software.

**Figure 5:**
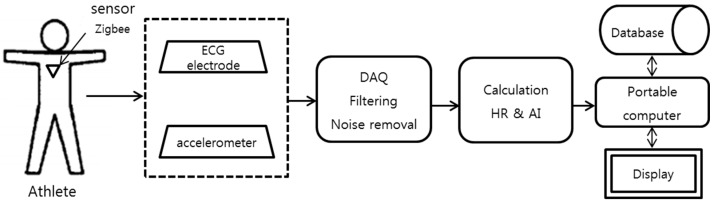
Schematic of the complete system (DAQ: Data Acquisition, AI: Agility Index).

The overall data processing is summarized as follows. First, the bio-signals from the ECG and 3-axis accelerometer sensors are acquired. Further, the signals are processed by converting the analog signal to a digital signal with low-pass and high-pass filters. Subsequently, the signals are wirelessly transmitted in real time from the sensor node to the receiver. Finally, the embedded analysis software provides the interface that receives and displays the monitoring signal in a personal computer. The data can be saved by date and time. The athlete training data can be listed on the calendar from a portable computer. The coach can easily check the athlete’s daily training status. The schematic of the system is shown in Figure 5.

### 3.1. Sensor Board

**Figure 6:**
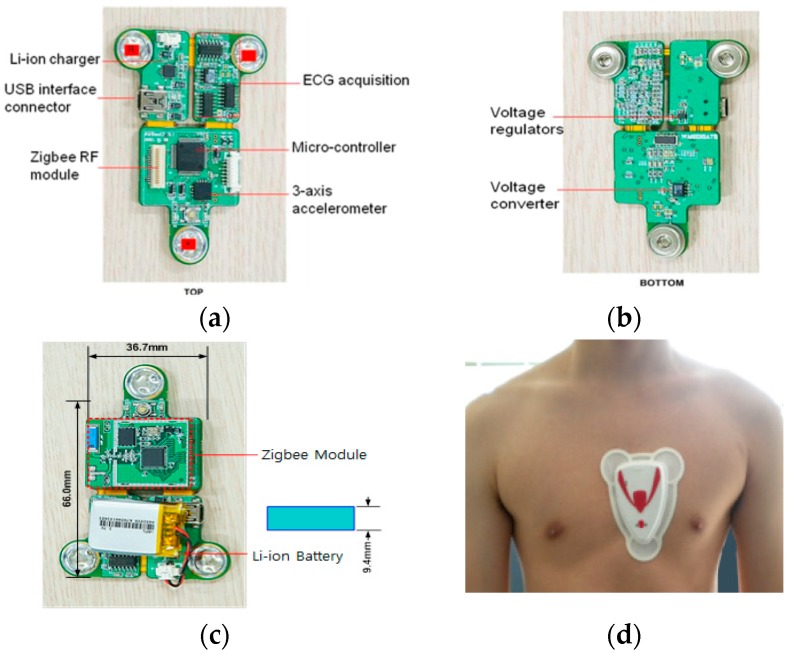
Sensor node: (**a**) top; (**b**) bottom; (**c**) packed with RF module and battery; (**d**) silicone-covered sensor node patched on athlete’s chest. USB = universal serial bus.

The sensor node comprises a triangular chip integrated with the ECG electrode, a ZigBee RF (Radio Frequency) communication module, a microcontroller, and a 3-axis accelerometer sensor. The top and bottom, packed with a battery and silicone-covered sensor node, are shown in Figure 6. The chip is wrapped in a soft rubber case that is comfortable and fits the contours of the body. The total weight of the sensor with the Li-ion battery is 20 g. The length of the sensor board is 66 mm and width is 36.7 mm. The sensor can be charged with a universal serial bus (USB) interface, and the maximal working time is eight hours. The communication distance is approximately 400 m in an open field. The specifications of the sensor node are shown in Table 1. The sensor is attached to the chest of an athlete to monitor the heart rate and agility index. The measured information is then sent to the host computer for further analysis.

### 3.2. Signal Acquisition and Processing

**Figure 7:**
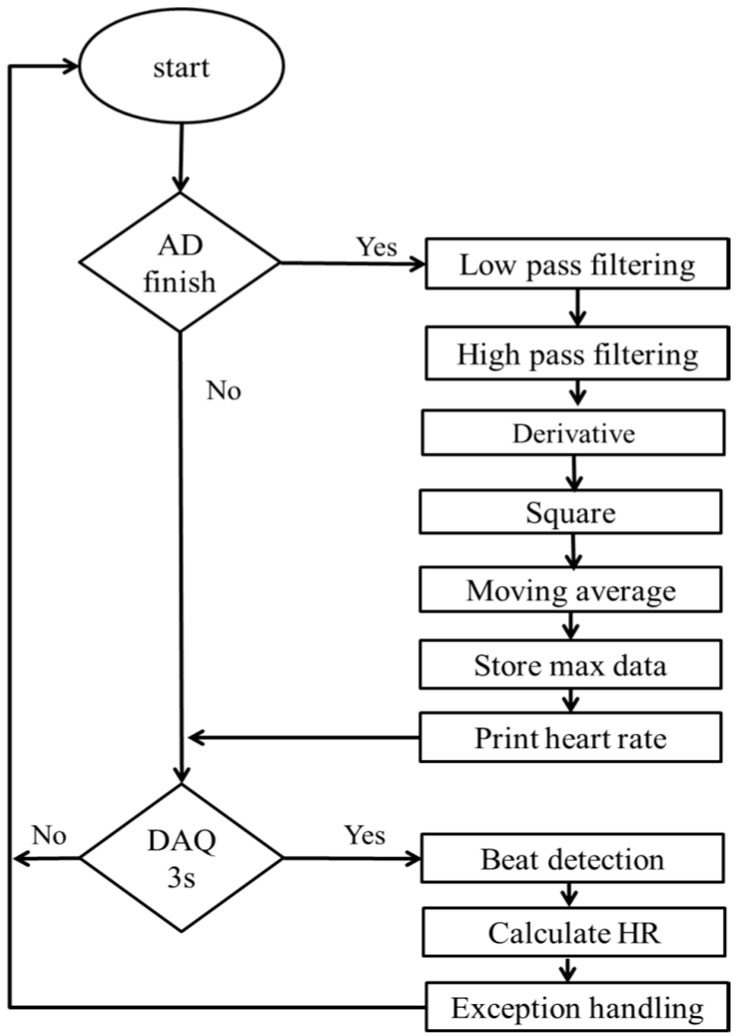
Flowchart for ECG signal processing.

The heart rate algorithm is implemented on the sensor board using the monitoring signals from the ECG electrodes. The sample data are analyzed using eight channels. The channels cover the frequencies from 1 to 50 Hz. After conversion to digital signals, signal acquisition commences from the low-pass to the high-pass filter. The signals are divided by time slots to find the voltage peak for the RR interval. Based on beat detection, the heart rate can be calculated and output to the personal computer. The heart rate signal acquisition and processing are shown in Figure 7.

The 3-axis accelerometer integrates all movement intensities into the agility index. Accelerometers are helpful for tracking human vibrations with a broad frequency range. In the calculation of the agility index, the *x*, *y*, and *z*-axis data are integrated into one output.

### 3.3. Transmission and Display

**Figure 8:**
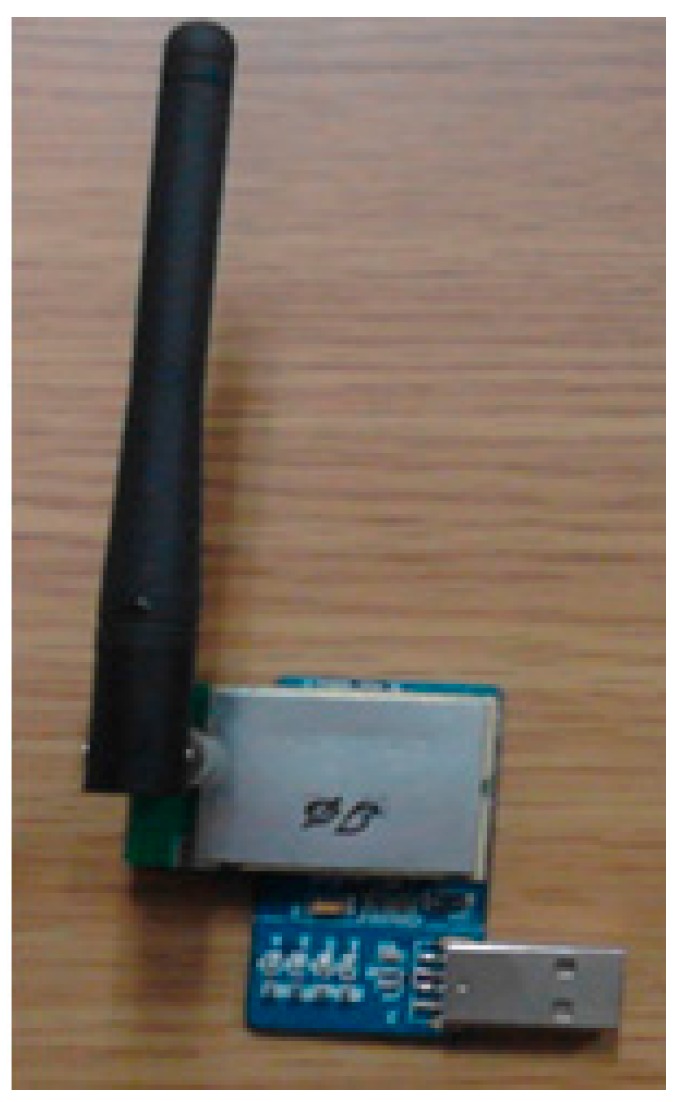
Receiver for the ZigBee communication protocol with a USB interface.

Wireless communication between the sensor and the receiver is achieved through a 2.4 GHz ZigBee RF-compatible module (LM2400, RadioPulse Inc., Seongnam-si, Korea). Figure 8 shows the chip antenna for receiving signals from the patch-type sensor. The biggest advantage of using a portable chip antenna is that it can be charged by a computer through the USB interface. The operation is easy for users as it enables plug-and-play operation. There is no need to consider the power consumption because the receiver already is part of a portable computer, which has long-lasting power storage, large data storage capacity, and processing capability for data analysis. The analysis system is developed in a Microsoft Visual C# environment.

**Figure 9:**
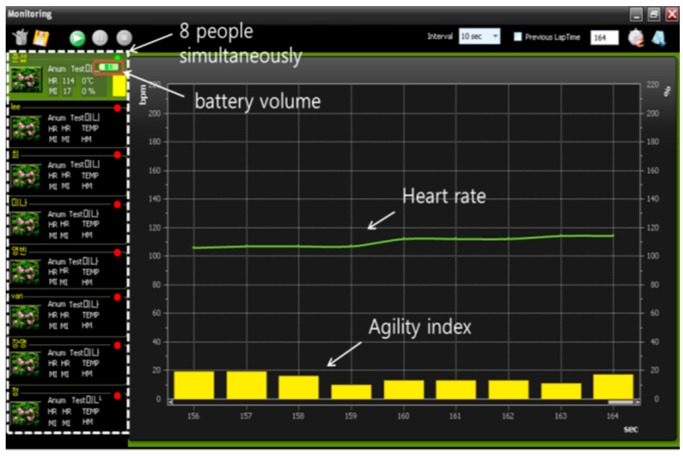
Screen shot of the monitoring interface.

Figure 9 shows a screen shot of the monitoring interface. The system can simultaneously monitor eight people and save the training data by date. The display of the battery volume for each sensor can prevent interruption during the physical training. With the support of analysis software, a coach can observe both the heart rate and agility index simultaneously in real time or save athletes’ information for further off-line analysis and evaluation. The coach can also evaluate athletic potential with the embedded evaluation software.

## 4. System Evaluation

### 4.1. Heart Rate Monitoring

To evaluate the performance of the proposed algorithm, tests were performed both in laboratory and field environments. In the laboratory environment, the accuracy of heart rate monitoring was verified by comparison with standard wired equipment (CASE Cardiac Assessment System, General Electric Company, Boston, MA, USA) [27]. The system was functionally evaluated in the field test. Twenty (ten males and ten females) participants were recruited from college for the two tests.

**Figure 10:**
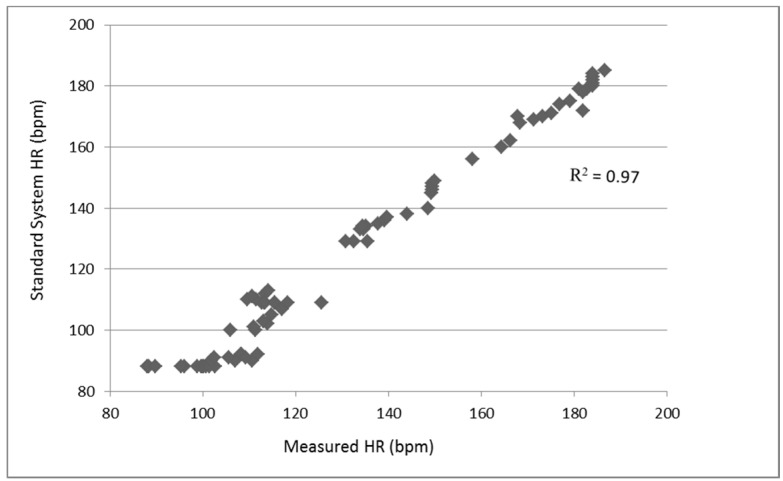
Heart rate correlation between proposed sensor and standard system.

The laboratory test was performed on a treadmill with speeds of 5, 10, and 15 km/h as well as 0 km/h in the rest state as shown in Table 2. This encompassed states from resting to walking and then running, which are basic activities for athletes. The participants were patched with the proposed sensors and heart rate measurement electrodes from the standard system. The duration of each test was 3 min for the steady measurement. Figure 10 shows the correlation between the proposed sensor and standard system. From the results, we can see that the exercise status exhibits greater accuracy compared to the rest status, which matches the activity pattern of athletes well. The average of the error is within 2%.

**Figure 11:**
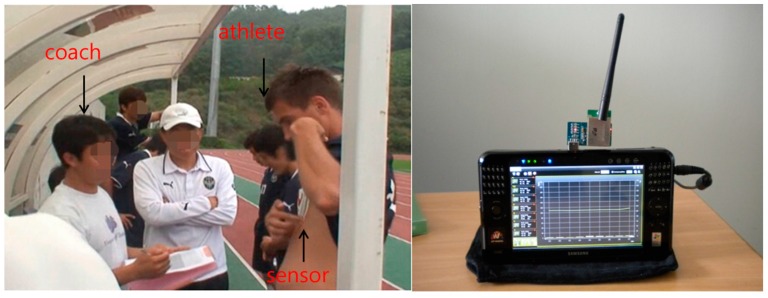
Experimental photo of field test.

**Figure 12:**
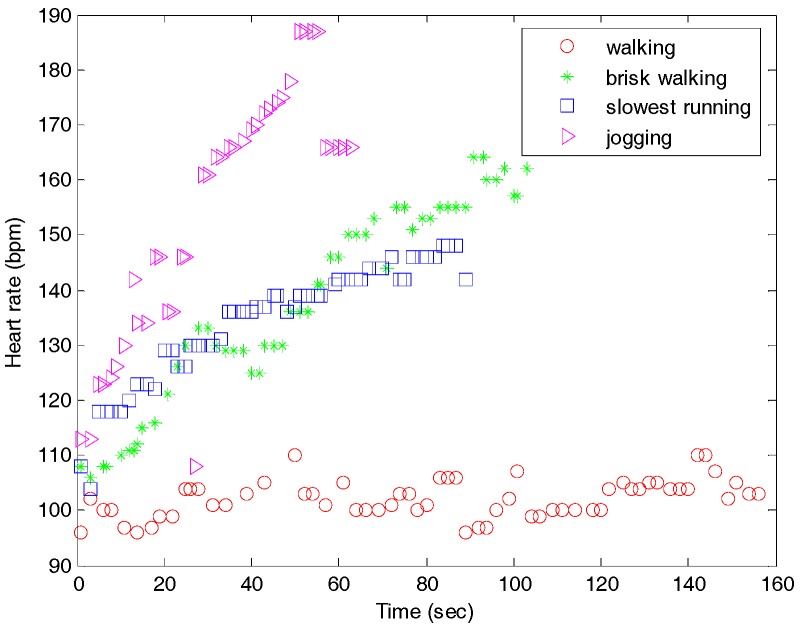
Heart rate monitoring during normal walking, brisk walking, slowest running, and jogging in an open field test.

The field test included normal walking, brisk walking, slowest running, and jogging. The design of this test demonstrated the monitoring capability as the distinguishable heart rate changed with a slight speed increase in real state tests. Each test course had the same conditions, and the distance was approximately 400 m in an open field as shown Figure 11. The procedure for the field test is shown in Table 3. The results of heart rate detection are shown in Figure 12. For the same distance, jogging took the least time with an increased slope for the heart rate. Walking resulted in a steady heart rate of 100 bpm. The slowest running produced almost similar heart rate levels for the entire period. At the start of the test, the slowest running produced a slightly higher heart rate than that in brisk walking. As time passed, brisk walking led to a higher heart rate than that for the slowest running. This is because slow running and quick walking exercises have equivalent energy consumptions.

### 4.2. Agility Detection

With high-intensity exercise, significant levels of noise in the human-sensor interface affect the signal measurements. To reduce the effects of noise, the zero-reference point, which is the output of the accelerometer under the non-movement condition, is utilized. The accelerometer output varies from the minimum (V_ddmin_) to the maximum (V_ddmax_) value. The zero value is near V_ddmin_ + (V_ddmax_ − V_ddmin_)/2. Values greater than the reference point represent positive values of acceleration, and the lower values represent deceleration. For agility index calculation, detecting the zero crossing is a very important process that extracts feature points of the acceleration signal. Zero crossing has been used in mathematics, engineering, and image processing. However, to the best of our knowledge, it has not been used in agility index calculation. In our study, first, we use the filters to reduce the noise. After that, we determine the feature points as maximum (V_ddmax_) and minimum (V_ddmin_) peak values from the output of the accelerometer under the non-movement condition. Finally, the agility index can be calculated by using the extracted feature points.

**Figure 13:**
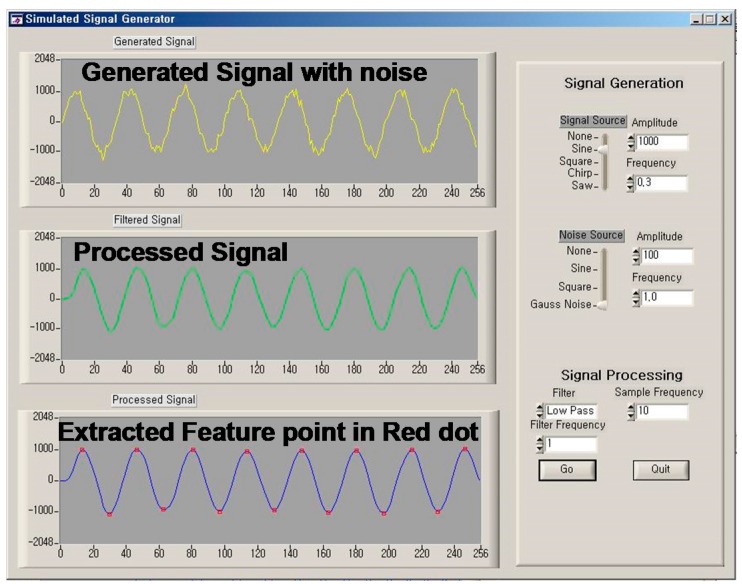
Screenshot of the simulated signal generator program for evaluating the feature point extraction.

This function was evaluated by using a simulated signal in an environment that included noise. A generator was used to simulate the signals with noise level control, as shown in Figure 13. The robust zero-crossing detector showed good extraction of feature points within a 1% error margin. With this procedure, the possibility of false value extraction owing to motion artifact noise could be reduced. The results of the algorithm’s performance tests show 99% correct detections on the simulated acceleration signal. The feature values can be calculated by using the extracted feature points provided by the robust zero-crossing detection.

**Figure 14:**
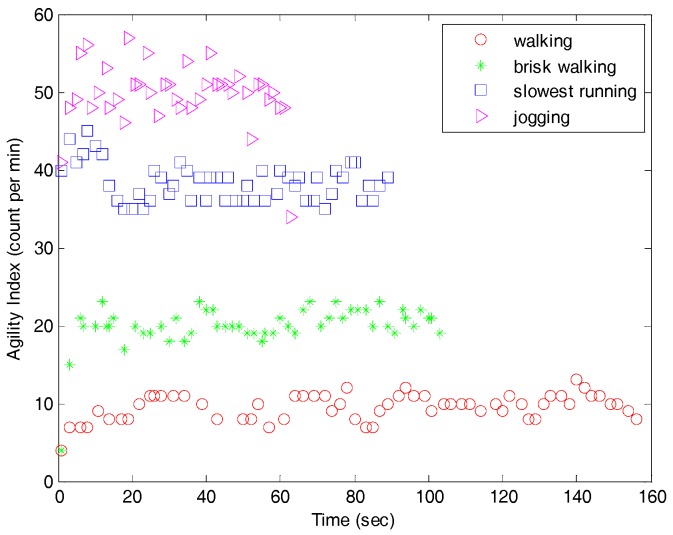
Agility index monitoring during normal walking, brisk walking, slowest running, and jogging in an open field test.

Figure 14 shows the detection of the agility index in the field test. The agility index integrates three directions into one output, which enables easy definition of the amount of exercise. Four different physical activities obviously show the distinguished value of the agility index, even though brisk walking and slowest running activities have similar actions.

## 5. Discussion of Athlete Evaluation

This section concludes with a discussion of the results of the system performance and the benefits of applying it to athletics training and evaluation. There are two commonly used evaluation methods.

(1) Fixed workload experiments
  Here, either one or multiple factors, such as workload intensity, time, and distance, are fixed during the experiments. In this study, the distance is fixed in the field test. Walking, jogging, brisk walking, and slowest running have the same distance of 400 m. The fixed workload experiments could respond to the stress reaction, and it indicates that it is capable of motion.
(2) Incremental workload experiments
  Here, the workload is gradually increased until the subjects feel exhausted. The test is for evaluating the ability of subjects when they enter the extreme state. The treadmill protocol is executed for the incremental experiments.

Heart rate measurement has many advantages in athletics training. In certain protocols, the heart rate and training intensity have a linear relationship. A high average heart rate in the training process indicates that the athlete has engaged in high-intensity training. Once the athlete overreaches or overtrains, the heart rate will increase at any running speed. This means that the training progress is not suitable for the athlete. By examining the daily heart rate records, the coach can easily find out the heart rate that athletes can sustain for different distances. This can help prevent the athletes from starting too fast in longer-distance races. Heart rate measurement can also help the coach select athletes. After implementing the same load-training program, the recovery heart rates can represent the athletes’ physical level [28]. Candidate athletes with lower recovery heart rates can be considered for selection. Traditional heart rate measurement in physical tests uses the fingertip and the pulse sensor, which is wired, and simultaneously measuring the heart rates of several people at the same time is a complicated undertaking. Our patch-type sensor can record the heart rate every second for eight people simultaneously, and the data can be downloaded to a personal computer for analysis. The battery enables continuous monitoring for eight hours. The sensor is lightweight and small, and a rubber case is selected for the best natural fit with the body curvature. Therefore, athletes do not suffer discomfort during training.

Table 4 shows the performance of several methods by various types of sensors. It is difficult to directly compare the results with existing methods in literature since most algorithms use different types of sensors (such as PPG, acoustic), different protocols (such as running, sleeping, resting) and with different subjects (females and males with different ages). The results of previous studies are validated by commercial standard systems. The comparison standard systems are also various. Despite these issues, compared to prior woks on HR monitoring, our algorithm showed competitive or superior performance. Our study used submaximal protocols with high intensity exercise on athletes that could generate more noise compare with previous studies.

Agility is detected by using three-dimensional accelerations. The agility index can help the coach control the training intensity based on the heart rate. The other important function is the sum of the agility index, which can be used in short-duration training to evaluate the athletes’ reaction. A high value for the sum of the agility index indicates a quick reaction; therefore, it can be applied in ball games, especially for football. We subjected ten football players to a zigzag run, 20-m shuttle run, burpee test, and sidestop test. These four tests are commonly used for training prescription [30], and each test was repeated ten times. The correlation coefficients compared with conventional agility were zigzag run (R^2^ = 0.90), 20-m shuttle run (R^2^ = 0.80), burpee test (R^2^ = 0.89), and sidestop test (R^2^ = 0.91).

**Figure 15:**
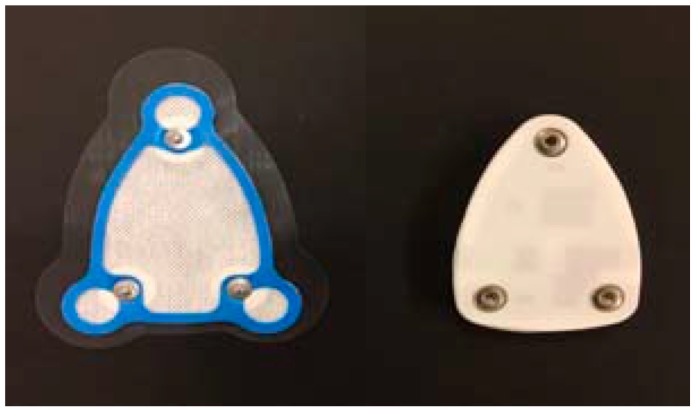
The adhesive coating between sensor and chest.

Finally, the peeling test was performed, which is important for a sports exercise sensor. In real-world training, athletes always perspire, especially in the chest area. Perspiration can cause the sensor electrodes to slip easily from the skin, and athletes can also feel uncomfortable, as is the case with most commercial sensors. The change in the electrical resistance of the skin can interfere with signal measurement. Our solution is to use a soft-skin adhesive coating. This material is made of spunlace fabric, which is resistant to water penetration and has good ventilation. The adhesive coating contains three dry Ag/AgCl electrodes without gel electrodes as shown in the left of Figure 15. The advantage of dry electrodes is elimination of allergic reaction or other forms of skin irritation [31]. The sensor board includes one-channel ECG which could accurately record cardiac potentials [32]. The electrode impedance problem has been raised in previous work [33,34]. In our studies, the electrodes are connected to a instrumentation amplifier, a notch filter, and a non-inverting amplifier to enhance the ECG signal [35]. The adhesive coating is attached to the chest and the sensor is attached to the coating, and the signals can be monitored. This design is tightly attached to the chest, which reduces the noise caused by motion artifacts. Furthermore, the strength of the adhesive was tested in all training programs, and there was no sensor drop during the entire training. Our experimental results can be summarized as follows:

(1) The small, lightweight patch-type sensor can wirelessly monitor the heart rate and agility simultaneously, which are two important factors for the selection and training of athletes.
(2) The system monitors the heart rate accurately compared with standard wired measurement systems.
(3) The agility is sensitive enough to distinguish between activities of similar intensities, such as slowest running and brisk walking.
(4) The peeling test was also performed to take into account a falling-off of the sensor due to perspiration. The average maximum adhesive strength under high-intensity training conditions was 0.863 kgf.

## 6. Conclusions

An algorithm for heart rate and agility index monitoring has been presented for athlete training and evaluation. The small, lightweight wireless sensor with a portable computer equipped with installed analysis software can be easily operated by the coach. We demonstrated key innovations in architectural and physical design such as (1) improved R-wave detection algorithms with a band-pass filter design to reduce the artifact noise from intensive exercise; (2) a robust zero-crossing algorithm for the agility index calculation; and (3) the case design, which uses rubber for the case and spunlace fabric coating for comfort during training. The system was tested both under laboratory and field environments to verify its accuracy and feasibility for real-world applications. Twenty participants were recruited from college. The heart rate accuracy was within 2% compared with a standard wired system (R^2^ = 0.97). The average correlation coefficient of agility was 0.9, which was tested with the commonly used training protocols. In comparison with state-of-the-art methods in the field, our proposed algorithm shows competitive results in various training tests, including walking and running at different speeds. In further research, the application of the system not only to athletic training but also in fields such as healthcare and human energy expenditure will be explored.

## Figures and Tables

**Table 1:** Specifications of the sensor node.

Components	Specification
Channel	8 ch
Resolution	12 bit
Sampling rate	200/s
Frequency bandwidth	1–50 Hz
Battery	Li-ion
Max HR	250/m
Power	3.3 V
Communication module	ZigBee
Communication distance	400 m
Microcontroller unit (MCU)	MSP430 (Texas Instruments, Dallas, TX, USA)
Electrode	Jumper setting available
Size	6 cm × 9 cm, 20 g

**Table 2:** Protocol for laboratory test.

Stage	Speed (km/h)	Duration (min)
1	0	3
2	5	3
3	10	3
4	15	3

**Table 3:** Procedure for field test.

Stage	Type	Distance (m)
1	Walking	400
2	Brisk walking	400
3	Slowest running	400
4	Jogging	400

**Table 4:** Comparison with other works in the literature.

Ref.	Subject	Sensor	Protocol	Position	Comparison with	Results
[11]	5 (two females and three males, aged 21.4 ± 0.5)	PPG	running 500 m	wrist	holter ECG (Cardy 303 pico, Suzuken Corp., Nagoya, Japan)	r = 0.98, SD = 8.7 bpm
[7]	10	acoustic	sleep in a pilot clinical study	neck	SOMNOscreen and PULSOX 300i pulse oximeter (Konica Minolta Sensing, Inc., Osaka, Japan)	Error rate 9.27 and 9.31%
[8]	10	PPG	ran for a maximum speed of 17 km/h	wrist	reference ECG	MAE (Mean Absolute Error) = 2.57%
[9]	12	PPG	fast running at the peak speed of 15 km/h	wrist	ground-truth	MAE = 2.34 beats-per-minute (BPM)
[10]	12 (male, aged 18~35)	PPG	not reported	wrist	ground-truth	1.77 ± 1.20 BPM (mean ± SD)
[29]	5 patients	Pan Tompkins	not reported	Android	Fluke PS400 ECG signal generator/simulator	Error rate 1.6% and 0.4%
this work	20 (ten males and ten females, aged 20~25)	patch-type sensor	submaximal protocol	chest	CASE Cardiac Assessment System	Error rate within 2%
